# Supplementary figures and images for: Golgi reassembly and stacking protein 65 downregulation is required for the anti-cancer effect of dihydromyricetin on human ovarian cancer cells
Source: PLoS One. 2019 Nov 26;14(11):e0225450. doi: 10.1371/journal.pone.0225450 (PMC6879129; doi:10.1371/journal.pone.0225450)

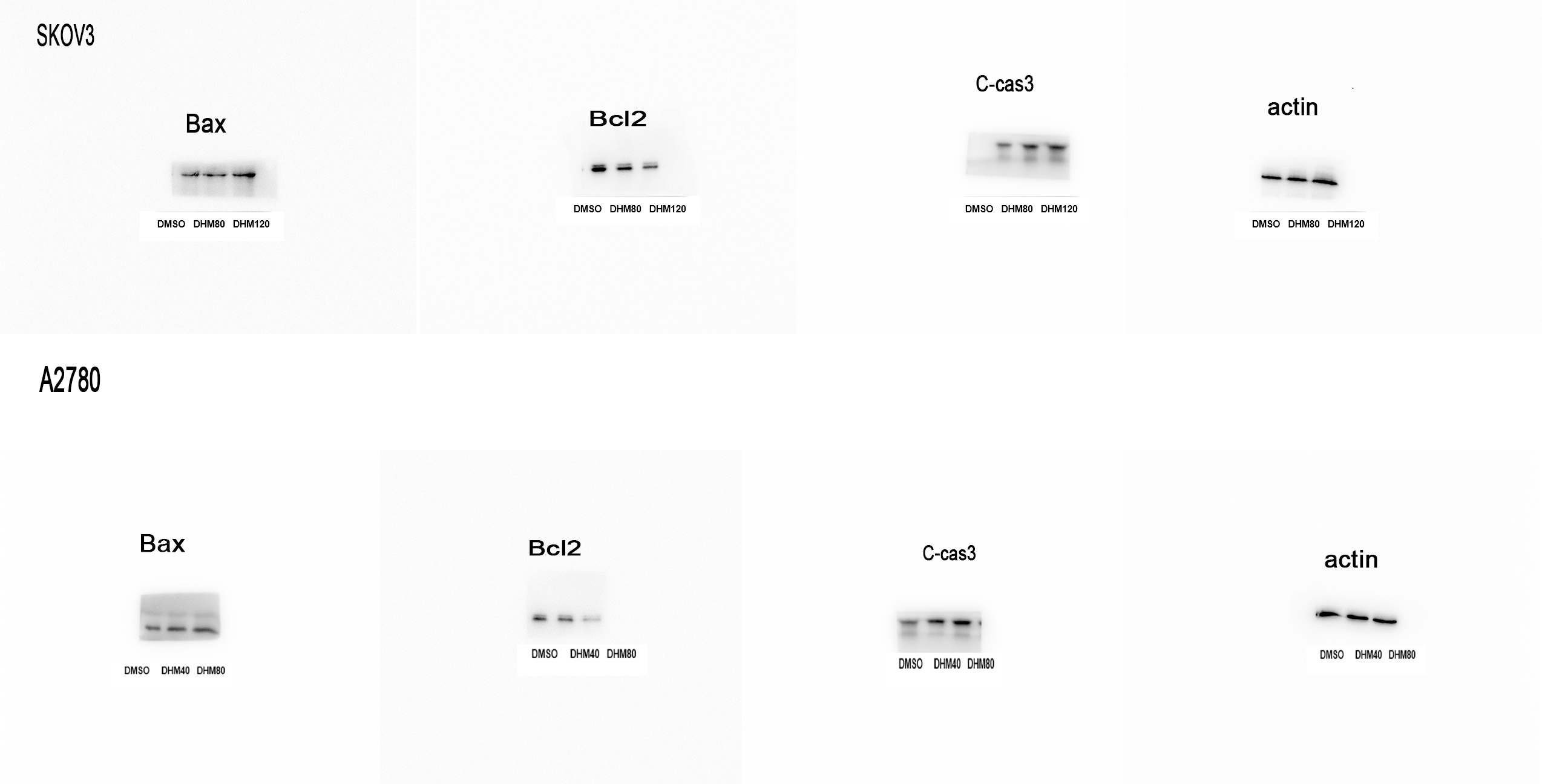

Supplement: S1 Fig — (TIF) [file pone.0225450.s001.tif]

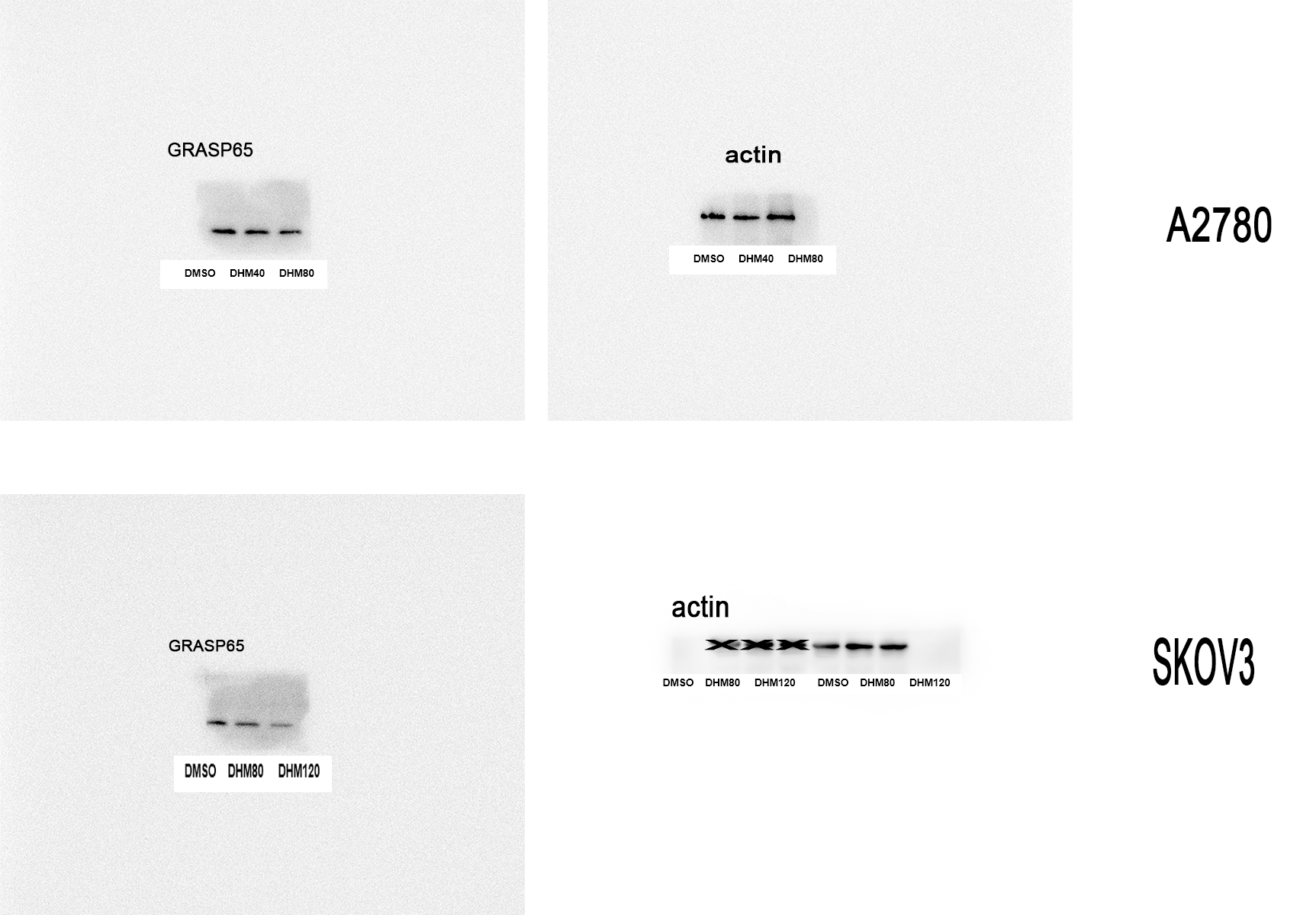

Supplement: S2 Fig — (TIF) [file pone.0225450.s002.tif]

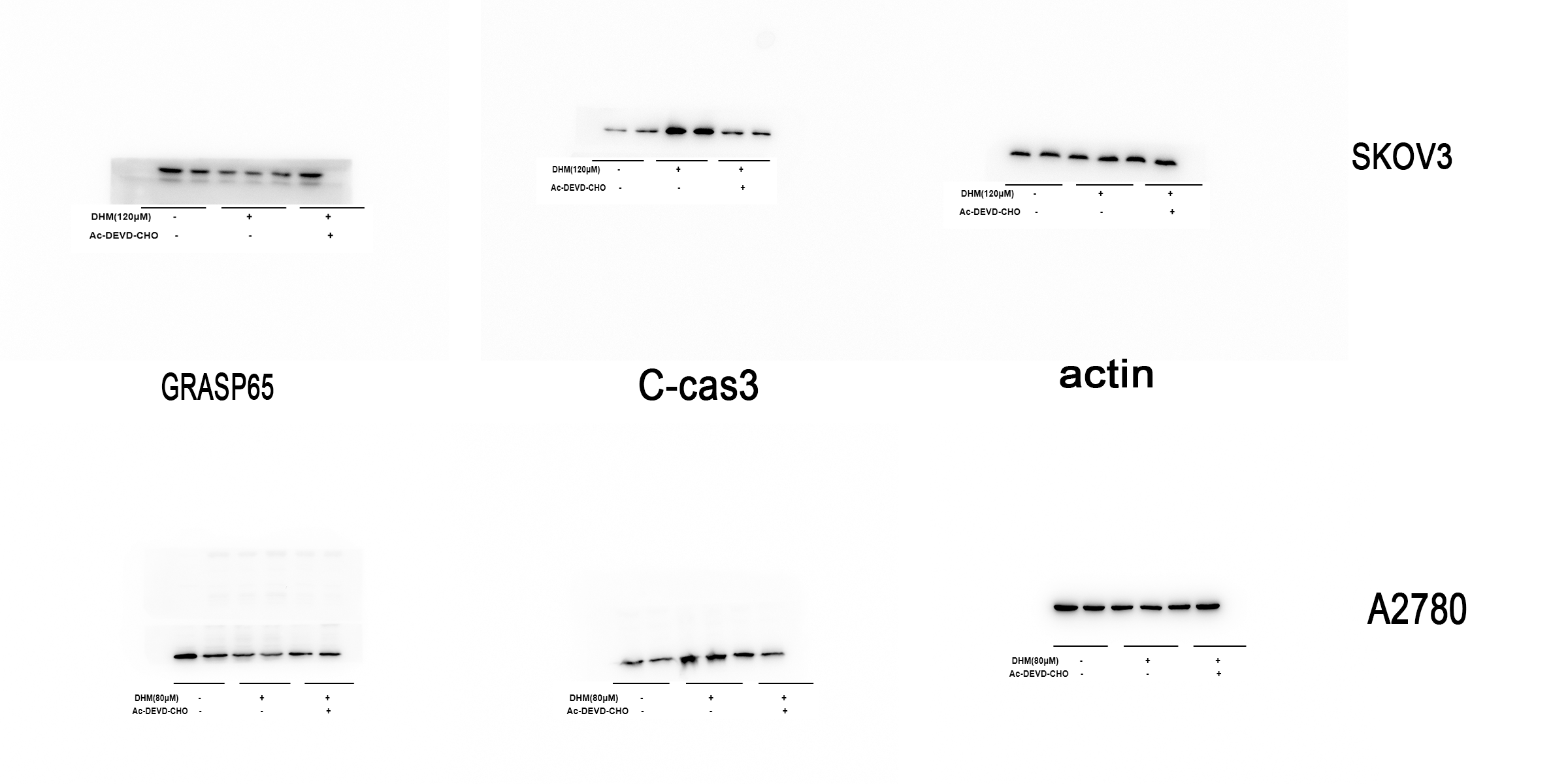

Supplement: S3 Fig — (TIF) [file pone.0225450.s003.tif]

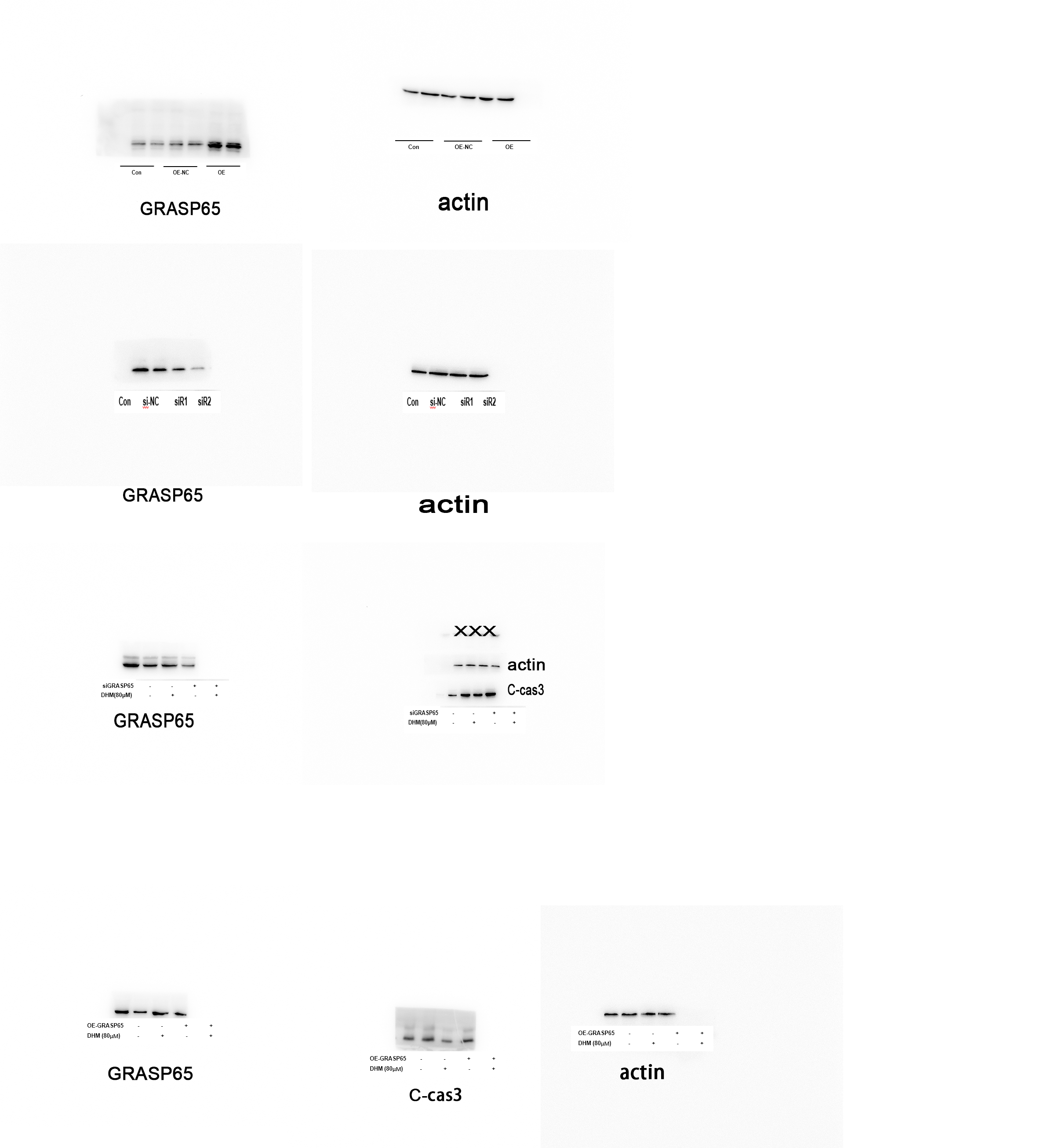

Supplement: S4 Fig — (TIF) [file pone.0225450.s004.tif]

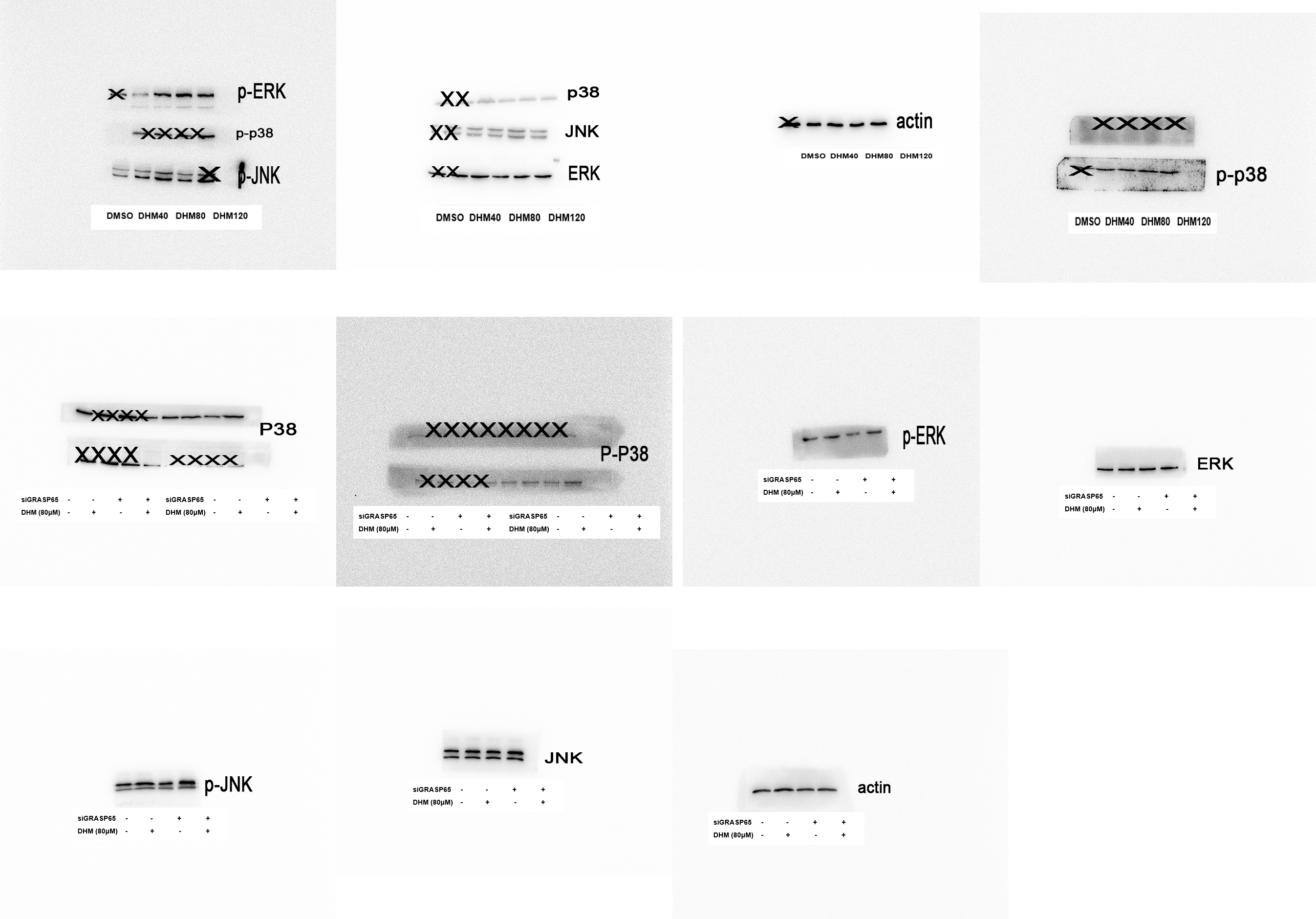

Supplement: S5 Fig — (TIF) [file pone.0225450.s005.tif]

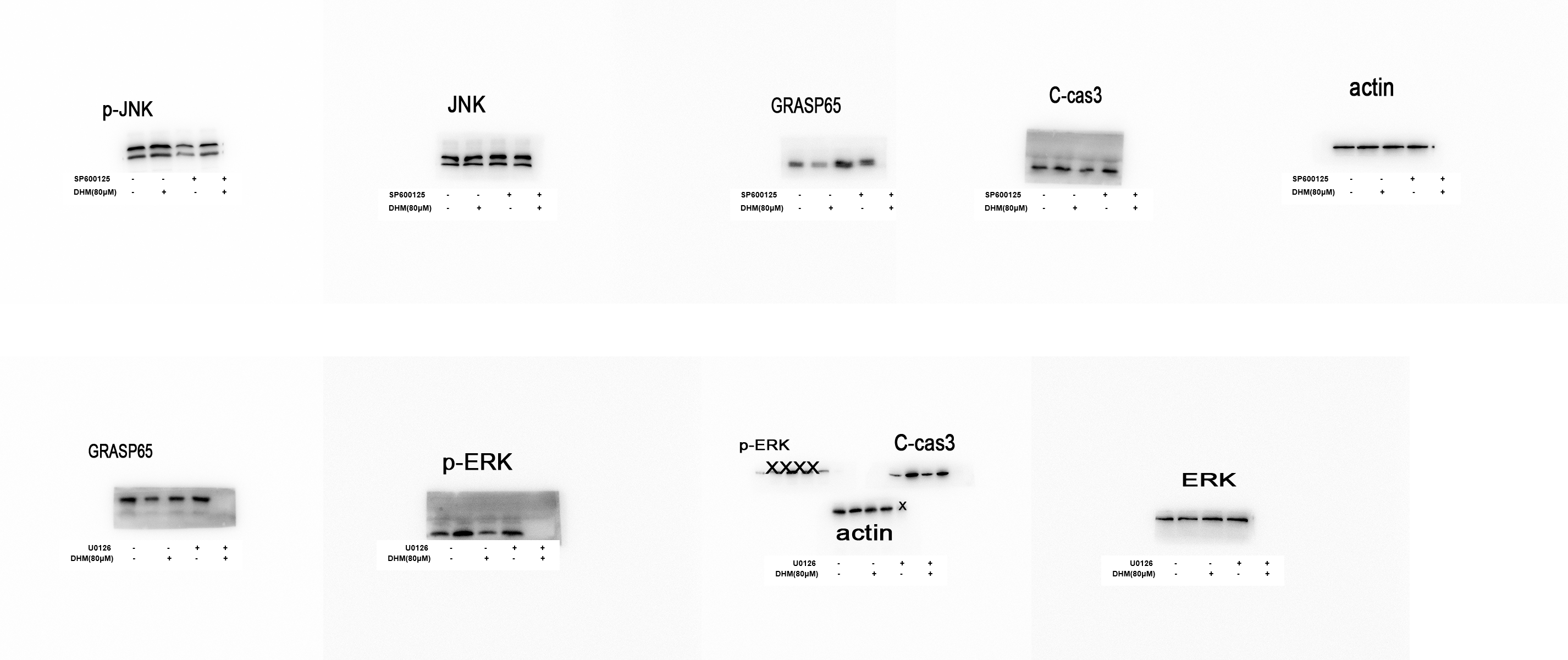

Supplement: S6 Fig — (TIF) [file pone.0225450.s006.tif]
